# Supplementary material for: Identification and functional analysis of non-coding regulatory small RNA FenSr3 in Bacillus amyloliquefaciens LPB-18
Source: PeerJ. 2023 May 15;11:e15236. doi: 10.7717/peerj.15236 (PMC10194069; doi:10.7717/peerj.15236)
Supplement: Supplemental Information 4 [file peerj-11-15236-s004.zip › KO/CK-vs-T1_map/map00900.html]

KEGG PATHWAY: Terpenoid backbone biosynthesis - Reference pathway


|  |  |
| --- | --- |
| **Terpenoid backbone biosynthesis - Reference pathway** |  |

[
Pathway menu
| Organism menu
| Pathway entry
| Show description
| User data mapping
]

Terpenoids, also known as isoprenoids, are a large class of natural products consisting of isoprene (C5) units. There are two biosynthetic pathways, the mevalonate pathway [MD:M00095] and the non-mevalonate pathway or the MEP/DOXP pathway [MD:M00096], for the terpenoid building blocks: isopentenyl diphosphate (IPP) and dimethylallyl diphosphate (DMAPP). The action of prenyltransferases then generates higher-order building blocks: geranyl diphosphate (GPP), farsenyl diphosphate (FPP), and geranylgeranyl diphosphate (GGPP), which are the precursors of monoterpenoids (C10), sesquiterpenoids (C15), and diterpenoids (C20), respectively. Condensation of these building blocks gives rise to the precursors of sterols (C30) and carotenoids (C40). The MEP/DOXP pathway is absent in higher animals and fungi, but in green plants the MEP/DOXP and mevalonate pathways co-exist in separate cellular compartments. The MEP/DOXP pathway, operating in the plastids, is responsible for the formation of essential oil monoterpenes and linalyl acetate, some sesquiterpenes, diterpenes, and carotenoids and phytol. The mevalonate pathway, operating in the cytosol, gives rise to triterpenes, sterols, and most sesquiterpenes.

|  |  |  |
| --- | --- | --- |
| Reference pathway | 184% 150% 122% 100% 82% 67% 55% | 图片下载 |
